# Heterogeneity of persisting symptoms after sport-related concussion (SRC): exploring symptom subtypes and patient subgroups

**DOI:** 10.1007/s00415-022-11448-6

**Published:** 2022-11-21

**Authors:** S. Langdon, E. Goedhart, M. Inklaar, J. Oosterlaan, M. Königs

**Affiliations:** 1 Emma Children’s Hospital, Amsterdam UMC, University of Amsterdam, Emma Neuroscience Group, Department of Pediatrics, Amsterdam Reproduction and Development Research Institute, Amsterdam, The Netherlands; 2 Football Medical Centre, Royal Netherlans Football Association (KNVB), Zeist, The Netherlands; 3 Emma Children’s Hospital, Amsterdam UMC, University of Amsterdam, Follow-Me Program and Emma Neuroscience Group, Department of Pediatrics, Amsterdam Reproduction and Development Research Institute, Amsterdam, The Netherlands

**Keywords:** Cluster analysis, Persistent concussion symptoms, Principal component analysis, Sport-related concussion

## Abstract

This study aimed to explore the heterogeneity of persisting symptoms after sport-related concussion (SRC). We examined the structure of symptom subtypes within 163 patients with SRC (M = 16.7 weeks post-injury). Subsequently, we investigated the existence of subgroups of patients based on comparable configuration of co-occurring symptom subtypes. To explore factors that may contribute to the emergence of SRC patient subgroups, subgroups were compared on pre-injury (i.e., demographics and medical history), personality (Severity Indices of Personality Problems Short Form) and SRC characteristics (i.e., history of prior concussions, loss of consciousness and post-traumatic amnesia). To investigate the relevance of SRC subgrouping for clinical outcome, subgroups were compared on symptom severity (Sport Concussion Assessment Tool 5). The results provide empirical evidence for the existence of symptom subtypes, characterized as a: neurocognitive, fatigue, emotional, migraine and vestibular-ocular symptom subtype in patients with persisting SRC. Study results also showed evidence for the existence of SRC subgroups of patients with a comparable configuration of co-occurring prevailing symptom subtypes, including a neurocognitive-migraine, fatigue, migraine-emotional and neurocognitive-emotional subgroup. The subgroups differed on pre-injury, personality and SRC characteristics, suggesting that these factors may contribute to the emergence of specific SRC patient subgroups. The subgroups also differed in the severity of persisting symptoms, highlighting the clinical relevance of SRC subgrouping. These results support the idea that patient subgroups with persisting SRC with a comparable pattern of co-occurring symptom subtypes exists, which may require targeted prognosis, clinical management and treatment to optimize recovery.

## Introduction

Worldwide, 50–60 million people suffer from traumatic brain injury (TBI) each year [1, 2], among which the majority (60–95%) have mild TBI (i.e., concussion) [3]. Sports injuries are among the most common causes (24–29%) of concussion [4]. Although the majority of patients with a concussion recover within 10–14 days, 10–15% of patients suffer from persisting symptoms (i.e., for more than 1 month) [5]. Sport-related concussions (SRCs) show great heterogeneity in terms of the emergence, evolution and recovery of symptoms and functional impairments over time [6–8]. To date, the sources of this variability are largely unknown and a general effective treatment for SRC is missing, which might be due to the heterogenetic nature of SRC. Moreover, prominent differences between patients challenge the development and application of clinical SRC management. Better understanding of the heterogeneity in SRC symptoms could importantly contribute to the development of more targeted and personalized care.

In recent research, the variability in SRC symptomatology has spurred a search into possible symptom subtypes of concussion. Early descriptions of SRC subtypes based on clinical observation [9] have recently been revised into consensus-driven descriptions of symptom subtypes in the acute timeframe after concussion (0–3 days post-injury), for which support was subsequently found in meta-analytic evidence [10]. In a meta-cluster analysis, we recently provided further support for delineation of SRC symptoms into subtypes within the typical course of SRC recovery (within 1 month post-injury) [11]. More specifically, the existing literature provides evidence for the existence of at least five symptom subtypes that are characterized by (i) migraine symptoms; (ii) cognitive and emotional symptoms (iii); cognitive and sleep symptoms; (iv) neurological symptoms; and (v) undefined feelings (i.e., “don’t feel right” and confusion). The literature also provides evidence for relations between symptom subtypes and clinical outcomes [11]. More specifically, symptoms relating to the migraine subtype were most strongly related to aspects of clinical outcome (e.g., prolonged recovery and cognitive deficits). Symptom subtyping may be informative with regard to the underlying cause(s) and prognosis of SRC, and may be useful to define optimal combinations of interventions in SRC management. Unfortunately, there is no research on the classification of symptom subtypes beyond the typical course of recovery, while targeted SRC management may be considered particularly crucial for the group of patients with persistent symptoms after SRC, where considerable negative impact on daily life and participation is at stake [12–14].

While symptom subtyping may contribute to a better understanding of the heterogeneity in persistent symptoms after SRC, symptom subtypes are known to overlap (e.g., certain symptoms contribute to multiple subtypes) and can co-occur within individuals (e.g., patients may experience symptoms from multiple subtypes at the same time) [10, 11]. This idea supports the classification of patients with persisting SRC into subgroups with a comparable configuration of co-occurring symptom subtypes (i.e., symptom subgroups). Symptom subgrouping has shown potential for combat-related concussions, with clear relevance to clinical outcome (i.e., return to duty) [15]. To our best knowledge, no study to date has investigated symptom subgroups among patients with persisting symptoms of concussion. In addition, exploring what determinants may contribute to the emergence of SRC symptom subgroups might potentially be relevant to provide insight in risk and protective factors that may pave the road to new treatment approaches.

The aim of the current study is to explore the heterogeneity of persisting symptoms after SRC. This study will investigate (1) the structure of symptom subtypes based on symptoms that tend to co-occur within patients, (2) the existence of SRC subgroups of patients, based on comparable configuration of co-occurring symptom subtypes, (3) the potential clinical relevance of SRC patients subgroups by exploring (a) whether pre-injury, personality and clinical characteristics may contribute to the emergence of SRC patient subgroups, and (b) whether SRC patient subgroups differ in terms of persisting symptom severity. This is the first study to explore SRC subgroups in patients with persisting symptoms after SRC. The results may contribute to a better understanding of the heterogeneity in SRCs, and may potentially facilitate more targeted and individualized management of patients with SRC to optimize recovery after SRC.

## Methods

### Participants

#### Sample

This study examined 163 patients with persisting symptoms after SRC. These patients were referred by a general practitioner to the Concussion Clinic at the Sports Medical Center of the Royal Netherlands Football Association (KNVB) between March 2018 and August 2020. This clinic offers concussion management for patients with persisting symptoms after a sport-related concussion, where persisting symptoms are defined by incomplete recovery of symptoms beyond 4 weeks post-injury [7]. The clinic focuses on optimization of recovery following SRC, not luxation of recovery in the chronic phase. Therefore, the clinic is available for patients that initiate treatment up to 9 months post-injury. For this study, the sample was confined to the adult age range (> 16 years). To increase homogeneity in terms of injury severity, patients with suspected moderate/severe TBI were excluded from this study, as reflected by self-reported loss of consciousness (LOC) > 30 min; and/or (2) anterograde post-traumatic amnesia (PTA) > 24 h [16].

### Measures

#### Demographics and background information

Data on demographic information (i.e., sex, age and socio-economic status) and medical history (i.e., pre-injury diagnosed neurological, psychiatric, personality or learning disorders) were obtained using a custom-made questionnaire. Socio-economic status (SES) was defined as the highest level of current or finalized education, ranging from 1 (no education) to 8 (postdoctoral education) [17].

#### SRC characteristics

Information on history of prior concussion, the type of sport at which SRC was sustained, and SRC severity (i.e., presence of LOC, PTA and vomiting) were collected using a custom-made questionnaire.

#### Symptoms of concussion

Symptoms of concussion were assessed using the symptom scale of the widely used Sport Concussion Assessment Tool 5 (SCAT-5), which consists of 22-items. Severity of symptoms was rated on a 7-point Likert scale, ranging from 0 (none) to 6 (severe). In addition, patients rated what percentage of normal they felt, on a scale ranging from 0 (feeling absolutely not normal) to 100 (feeling completely normal). Each individual item score, the total score and the normality score were used for the current analysis. The SCAT-5 has shown excellent reliability (α = 0.95) [18].

#### Personality structure

Personality characteristics were measured using the Severity Indices of Personality Problems Short Form (SIPP-SF), assessing the following domains: self-control, identity integration, responsibility, relational capacities and social concordance. The SIPP-SF consists of 60 items rated on a 4-point Likert scale, ranging from 1 (fully disagree) to 4 (fully agree). Lower scores represent more prominent characteristics of personality disorders. The total SIPP-SF score and the score for each domain were used for the current analysis. The SIPP has adequate internal consistency reliability (Cronbach’s alpha = 0.80) [19].

### Procedure

Structured prospective clinical measurements were obtained using online questionnaires that were sent out 1 week prior to the intake appointment to the clinic. Patients provided informed consent for re-use of clinical data for scientific studies. The medical ethical committee of the Amsterdam UMC (location AMC) approved this study (W18_045).

### Statistical analysis

All analyses were performed using R studio (v. 1.2.1) [20]. All dependent variables were screened for outliers by using box-plots. Outliers (> M + 2SD) were subsequently rescaled using Winsorizing with the ‘Desctools’ package in R [21]. In addition, dependent measures that were not normally distributed were subjected to van der Waerden transformation, using the ‘BestNormalize’ package in R [22]. Missing data (< 1% on all measures) were imputed by multiple imputation using the ‘MICE’ package in R [23, 24].

#### Symptom subtypes

To identify symptom subtypes, a principal component analysis with varimax rotation of the 22 SCAT symptoms was performed using the ‘psych’ package in R [25]. The number of components to extract was determined by the cumulative proportion of variance explained (R^2^ ~  > 60%) [26]. Subsequently, each extracted component was considered to represent a symptom subtype, which was labeled according to the set of symptoms that made the strongest contribution to the component in terms of effect size (*ƞ*^2^ > 0.4).

To control for differences in total symptom severity between patients (reflected by the total SCAT score), we calculated dominance scores for each symptom subtype. Therefore, each individual symptom subtype score was transformed to a z-score, with higher scores reflecting higher symptom severity. Second, individual dominance scores (%) were calculated for each symptom subtype by dividing the individual z-score on each symptom subtype by the sum of z-scores for all symptom subtypes of that individual. As such, the dominance score reflects the relative contribution of a given symptom subtype to the general symptom burden of each individual.

#### SRC patient subgroups

To identify SRC patient subgroups, a cluster analysis (*k*-means clustering) on the dominance scores of the identified symptom subtypes was conducted, using the ‘NbClust’ package in R [27]. To investigate the identity of the determined SRC patient subgroups, we compared each subgroup to the other subgroups combined on the dominance scores of the symptom subtypes using *t*-testing. The observed group differences and effect size (Cohen’s *d* > 0.50) [28] were used to label subgroups based on the configuration of prevailing symptom subtypes that were found characteristic of a subgroup.

#### Pre-injury, personality and SRC characteristics

To explore factors that may contribute to the emergence of SRC patient subgroups, each subgroup was compared to all other subgroups on pre-injury characteristics (i.e., sex, age, SES, psychiatric and learning disorders), personality characteristics (i.e., total SIPP-SF score) and SRC characteristics (i.e., history of prior concussions, LOC and PTA). To guard against type-1 errors, significant subgroup differences on total SIPP-SF scores were followed-up by subgroup comparisons at the level of the five subscale scores.

#### Symptom severity

To investigate total severity of persisting symptoms, each symptom subgroup was also compared to all other subgroups combined on the total SCAT score and SCAT normality score, using independent *t*-tests.

All statistical testing was two-sided at α = 0.05 and effect sizes of group differences were calculated in terms of Cohen’s *d* for continuous dependent variables and odds ratios (OR) for binary dependent variables [28].

## Results

### Background information

Demographic information, SRC characteristics and medical history of 163 patients with persistent symptoms after concussion are displayed in Table 1.

**Table 1:** Demographic and injury-related information

	*N* = 163
Demographic information	
Males, *n* (%)	69 (42)
Age at intake in years, M (SD)	29.2 (10.4)
SES, M (SD)	5.9 (1.2)
SRC characteristics	
History of prior concussion, *n* (%)	78 (48)
Type of sport related to injury, *n* (%)	
Soccer	54 (33)
Hockey	22 (14)
Cycling	15 (9)
Other	72 (44)
Time since injury in weeks, M (SD), range	16.7 (7.7), 4.6–35.9
Loss of consciousness, *n* (%)	38 (23)
Anterograde posttraumatic amnesia, *n* (%)	45 (28)
Vomiting, *n* (%)	13 (8)
Medical history	
Neurological disorder, *n* (%)	4 (3)
Migraine, *n* (%)	2 (2)
Stroke, *n* (%)	1 (1)
Other, *n* (%)	1 (1)
Psychiatric disorder, *n* (%)	11 (7)
Depression, *n* (%)	4 (3)
ADHD, *n* (%)	3 (2)
Anxiety, *n* (%)	1 (1)
OCD, *n* (%)	1 (1)
Personality disorder, *n* (%)	1 (1)
Eating disorder, *n* (%)	1 (1)
Learning disorder, *n* (%)	16 (10)
Dyslexia, *n* (%)	16 (10)

*ADHD* attention-deficit/hyperactivity disorder, *M* mean, *OCD* obsessive compulsive disorder, *SD* standard deviation, *SES* socio-economic status

### Symptom subtypes

**Fig. 1:**
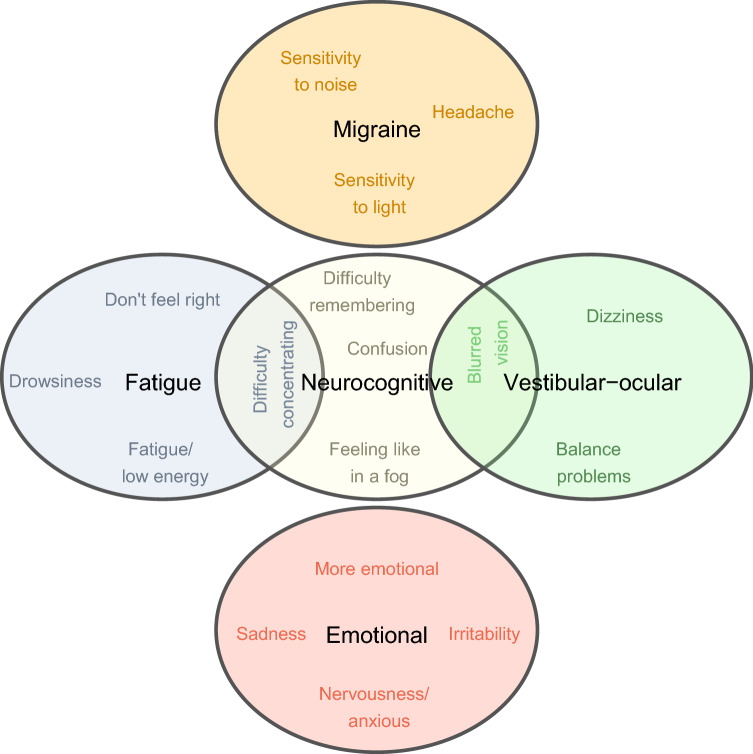
Visual representation of the identified SRC symptom subtypes

To explore the existence of symptom subtypes, a principal component analysis was performed on the 22-items of the SCAT-5. The results supported a component solution discriminating between five clusters (i.e., symptom subtypes), accounting for 59.8% of the variance. According to the set of the symptoms that made the strongest contribution to each cluster, the symptom subtypes were labelled as follows (see Table 2): (1) *neurocognitive* subtype: 14.0% of variance, 5 items; (2) *fatigue* subtype: 13.2% of variance, 4 items; (3) *emotional* subtype: 12.9% of variance, 4 items; (4) *migraine* subtype: 11.1% of variance, 3 items; and (5) *vestibular-ocular* subtype: 8.6% of variance, 3 items. A visual representation of the identified symptom subtypes and their relations to specific symptoms is displayed in Fig. 1.

**Table 2:** Symptom subtypes based on principal component analysis on symptom items

	Symptom subtypes				
Symptom	Neurocognitive	Fatigue	Emotional	Migraine	Vestibular-ocular
Headache	0.104	0.124		0.792*	
Pressure in head	0.291	0.327	0.147	0.136	0.169
Neck pain			0.131	0.136	0.140
Nausea or vomiting		0.208			
Dizziness	0.244	0.194	0.228	− 0.115	0.745*
Blurred vision	0.456*				0.480*
Balance problems				0.173	0.836*
Sensitivity to light	0.180		0.252	0.592*	0.270
Sensitivity to noise	0.123	0.188	0.308	0.651*	
Feeling slowed down	0.316	0.377	0.201	0.133	0.155
Feeling like in a fog	0.722*	0.370	0.211	0.178	0.187
Don’t feel right	0.279	0.469*	0.337	0.355	0.309
Difficulty concentrating	0.661*	0.404*	0.155	0.321	0.151
Difficulty remembering	0.783*	0.247		0.274	
Fatigue or low energy	0.280	0.780*	0.214	0.134	
Confusion	0.748*	0.248	0.220		0.154
Drowsiness	0.218	0.798*		0.122	
More emotional	0.272	0.151	0.796*	0.183	
Irritability	0.367		0.528*	0.352	
Sadness	0.138	0.162	0.863*	0.168	
Nervousness or anxious		0.169	0.759*		0.231
Trouble falling asleep		0.285	0.146		

Item loading meeting criteria for component labeling are displayed in bold and marked with asterisks

### SRC patient subgroups

**Fig. 2:**
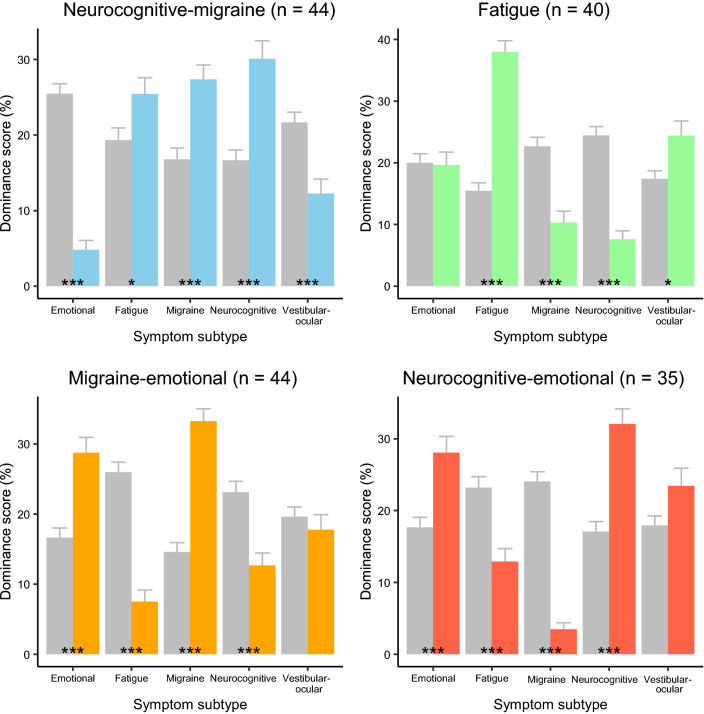
Configuration of symptom subtypes within SRC patient subgroups. Error bars represent standard error. *p < 0.05. ***p < 0.001

To identify SRC patient subgroups with a comparable configuration of prevailing symptom subtypes, a cluster analysis was conducted. Based on the screen plot, we choose to extract a clinically manageable number of four clusters of patients, i.e., the patient subgroups. Figure 2 shows the comparison of each subgroup to the other subgroups combined on the dominance scores. The first subgroup (*n* = 44) was characterized by higher dominance scores on neurocognitive symptoms (*t*(72.9) = − 4.8, *p* < 0.001, *d* = 0.87), migraine symptoms (*t*(99.4) = − 4.3, *p* < 0.001, *d* = 0.72) and fatigue symptoms (t(92.6) = − 2.3, *p* = 0.026, *d* = 0.38) as compared to the other subgroups. The first subgroup was therefore labeled as the *neurocognitive-migraine* subgroup, which also displayed lower dominance scores on emotional symptoms (*t*(131.7) = 11.5, *p* < 0.001, *d* = 1.77) and vestibular-ocular symptoms (*t*(89.5) = 4.0, *p* < 0.001, *d* = 0.67) than the other subgroups. The second subgroup (*n* = 40) showed higher dominance scores on fatigue symptoms (*t*(81.4) = − 10.0, *p* < 0.001, *d* = 1.72) and vestibular-ocular symptoms (*t*(83.9) = − 2.6, *p* < 0.013, *d* = 0.47) than the other subgroups. The second subgroup was labeled as the *fatigue* subgroup, which also displayed lower dominance scores on migraine symptoms (*t*(91.7) = 5.2, *p* < 0.001, *d* = 0.87) and neurocognitive symptoms (*t*(128.0) = 8.5, *p* < 0.001, *d* = 1.30) than the other subgroups. The third subgroup (*n* = 44) was characterized by higher dominance scores on migraine symptoms (*t*(89.9) = -8.5, *p* < 0.001, *d* = 1.41) and emotional symptoms (*t*(79.7) = − 4.7, *p* < 0.001, *d* = 0.82) as compared to the other subgroups. Therefore, the third subgroup was labeled as the *migraine-emotional* subgroup, which also displayed lower dominance scores on neurocognitive symptoms (*t*(109.3) = 4.4, *p* < 0.001, *d* = 0.71) and fatigue symptoms (*t*(111.7) = 8.4, *p* < 0.001, *d* = 1.35) than the other subgroups. The last subgroup (*n* = 35) showed higher dominance scores on neurocognitive symptoms (*t*(66.3) = − 5.9, *p* < 0.001, *d* = 1.05) and emotional symptoms (*t*(62.6) = − 3.9, *p* < 0.001, *d* = 0.71) than the other subgroups. The last subgroup was therefore labeled as the *neurocognitive-emotional* subgroup and also showed lower dominance scores on fatigue symptoms (*t*(89.3) = 4.3, *p* < 0.001, *d* = 0.71) and migraine symptoms (*t*(152.1) = 12.6, *p* < 0.001, *d* = 1.80) than the other subgroups.

### Clinical relevance of SRC patient subgroups

To explore subgroup differences, each patient subgroup was compared to the other subgroups combined on pre-injury characteristics (i.e., demographic information and medical history), personality characteristics, SRC characteristics and symptom severity (see Table 3).

**Table 3:** Analysis of pre-injury characteristics, personality characteristics, SRC characteristics and symptom severity in the SRC patient subgroups

	Subgroup				Contrasts		
	Neurocognitive-migraine (1)	Fatigue (2)	Migraine-emotional (3)	Neurocognitive-emotional (4)		*p*	*Effect size*
*N*	44	40	44	35			
Pre-injury characteristics							
Males, *n* (%)	16 (36)	19 (48)	17 (39)	17 (49)	NS		
Age at intake in y, M (SD)	28.1 (11.5)	29.0 (10.2)	30.8 (10.3)	28.6 (9.3)	NS		
SES, M (SD)	5.5 (1.2)	6.1 (1.2)	6.2 (0.9)	5.9 (1.1)	1 < other	0.007	*d* = 0.50
					3 > other	0.045	*d* = 0.33
Psychiatric disorder, *n* (%)	4 (9)	3 (8)	3 (7)	1 (3)	NS		
Learning disorder, *n* (%)	7 (16)	2 (5)	1 (2)	6 (17)	NS		
Personality characteristics							
Total SIPP-SF score, M (SD)	214 (16.3)	218 (17.5)	209 (20.7)	204 (21.2)	2 > other	0.014	*d* = 0.48
					4 < other	0.028	*d* = 0.45
SRC characteristics							
Prior concussion, *n* (%)	22 (50)	15 (38)	22 (50)	19 (54)	NS		
Loss of consciousness, *n* (%)	16 (36)	7 (18)	8 (18)	7 (20)	1 > other	0.029	OR = 1.32
Anterograde PTA, *n* (%)	14 (32)	10 (25)	10 (23)	11 (31)	NS		
Symptom severity							
Total SCAT score, M (SD)	46.8 (19.4)	36.5 (22.5)	37.8 (19.6)	36.3 (22.8)	1 > other	0.006	*d* = 0.48
SCAT normality score, M (SD)	54.1 (18.5)	58.1 (22.6)	52.5 (20.9)	70.0 (18.7)	3 < other	0.042	*d* = 0.37
					4 > other	< 0.001	*d* = 0.77

*M* mean, *NS* not significant, *PTA* posttraumatic amnesia, *SCAT* sport concussion assessment tool 5, *SD* standard deviation, *SDQ* strength and difficulties questionnaire, *SES* socio-economic status, *SIPP*-*SF* severity indices of personality problems short form, *w* weeks, *y* years

#### Pre-injury characteristics

The patient subgroups did not differ on premorbid characteristics (− 1.9 ≤ *ts* ≤ 2.8, *ps* ≥ 0.07), with the single exception of SES. More specifically, the *neurocognitive-migraine* subgroup had lower SES than the other subgroups (*t*(68.9) = 2.8, *p* = 0.007, *d* = 0.50), while the *emotional-migraine* subgroup had higher SES than the other subgroups (*t*(107.7) = -2.0, *p* = 0.045, *d* = 0.33).

#### Personality characteristics

With regard to personality characteristics, the *neurocognitive-emotional* subgroup showed lower scores on the SIPP-SF (*t*(52.0) = 2.3, *p* = 0.028, *d* = 0.45), indicating that SRC patients with a combination of predominating neurocognitive and emotional symptoms show more prominent characteristics of personality disorders. Follow-up analysis at the level of SIPP-SF subscale scores, showed that the *neurocognitive-emotional* subgroup had lower responsibility domain scores (*t*(51.1) = 2.1, *p* = 0.037, *d* = 0.44), indicative of poorer ability to set realistic goals and achieve these goals as compared to other SRC patient subgroups. Furthermore, we found that the *fatigue* subgroup showed higher scores on the SIPP-SF as compared to the other subgroups (*t*(63.5) = − 2.5, *p* = 0.014, *d* = 0.48). Subsequent follow-up analyses on SIPP-SF subscale scores, showed that self-control (*t*(64.2) = − 2.1, *p* = 0.042, *d* = 0.27), relational capacities (*t*(74.2) = − 2.4, *p* = 0.019, *d* = 0.50) and social concordance domain scores (*t*(71.2) = − 2.3, *p* = 0.023, *d* = 0.42) were higher for the *fatigue* subgroup than other subgroups. These results indicate that patients with prevailing fatigue-related symptoms have less prominent characteristics of personality problems in general and are more likely to better tolerate, use and control emotions and impulses (self-control), genuinely care about others (relational capacities) and value someone’s identity and work with others (social concordance) than other SRC patients.

#### SRC characteristics

In terms of SRC severity, the frequency of LOC in the *neurocognitive-migraine* subgroup was higher than in the other subgroups (χ^2^ (1, *N* = 163) = 4.8, *p* = 0.029), indicating that SRC patients with a combination of predominating neurocognitive and migraine related symptoms are more likely to have experienced LOC post-injury than other SRC patients. No other differences were observed on SRC characteristics (i.e., prior concussion and PTA; χ^2^s ≤ 1.8, *ps* ≥ 0.180).

#### Symptom severity

**Fig. 3:**
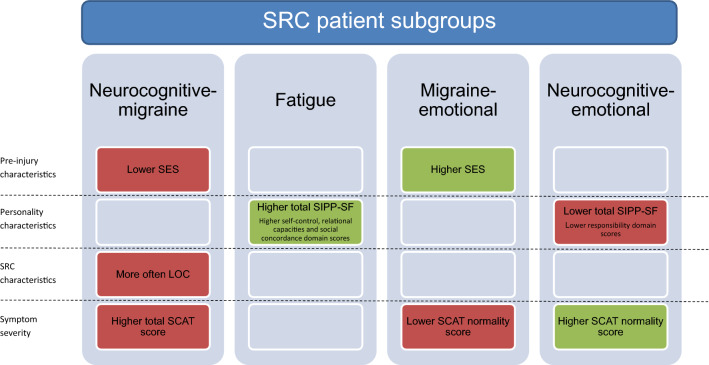
Overview of significant group differences between SRC patient subgroups on pre-injury characteristics, personality characteristics, SRC characteristics and symptom severity. Color-coding refers to worse (i.e., red) or better (i.e., green) scores/associations. *LOC* loss of consciousness; *SCAT* sport concussion assessment tool 5; *SES* socio-economic status; *SIPP-SF* severity indices of personality problems short form

Regarding the severity of persisting symptoms, the *neurocognitive-migraine* subgroup had higher total SCAT scores than the other subgroups (*t*(84.0) = − 2.8, *p* = 0.006, *d* = 0.48). In addition, the *migraine-emotional* group showed a lower normality score as compared to the other subgroups (*t*(77.1) = 2.1, *p* = 0.042, *d* = 0.37), while a higher normality score was observed in the *neurocognitive-emotional* group than the other subgroups (*t*(58.8) = − 4.2, *p* < 0.001, *d* = 0.77). The results indicate that, on the one hand, SRC patients with a combination of predominating neurocognitive and migraine symptoms experience higher symptom severity and SRC patients with a combination of prevailing migraine and emotional related symptoms feel less normal post-injury than other SRC patients. On the other hand, SRC patients with a combination of dominant neurocognitive and emotional related symptoms feel more normal following SRC than other SRC patients. An overview of the reported subgroup differences is displayed in Fig. 3.

## Discussion

This study aimed to explore the heterogeneity of persisting symptoms in a large sample of patients with sport-related concussion (SRC). The results indicate that persisting symptoms cluster within patients into distinct symptom subtypes. Furthermore, we present evidence for the existence of SRC subgroups of patients with a comparable configuration of co-occurring symptom subtypes. These SRC patient subgroups differ on pre-injury, personality and SRC characteristics, suggesting that complex interplay between such factors may contribute to the emergence of specific SRC patient subgroups. The SRC patient subgroups also differed in symptom severity, highlighting the relevance of SRC subgrouping for clinical outcome. Together, the findings of this study provide a framework for a better understanding of the distinct heterogeneity in SRC symptomatology, which may pave the way for the development of more personalized and targeted treatment of persisting symptoms after SRC.

The current study used a data-driven approach to provide empirical evidence for the existence of five subtypes of persisting symptoms after SRC, including a *neurocognitive*, a *fatigue*, an *emotional*, a *migraine* and a *vestibular-ocular* symptom subtype. These results align with previous work into the variability in SRC symptomatology, showing some overlap with acute symptom subtypes as established in meta analytic reviews [10, 11]. While previous work found evidence for symptom subtypes within the typical course of recovery, this study extends the existing literature by describing symptom subtypes beyond this phase (> 4 weeks post-injury; *M* = 16.7 weeks post-injury), characterizing long-lasting SRC. As compared to the previously determined acute symptom subtypes, vestibular and ocular symptoms seem to cluster together in the persisting subtypes, as do neurocognitive and neurological symptoms. These differences may reflect the evolution of symptom clustering over time since injury [7, 29].

Examination of systematic co-occurrence of symptom subtypes within patients further revealed evidence for the existence of distinct subgroups of SRC patient. Patients with a combination of predominating neurocognitive and migraine symptoms (*neurocognitive-migraine* subgroup) differentiated from other patients based on pre-injury characteristics (i.e., lower SES), SRC characteristics (higher incidence of LOC) and symptom severity (i.e., more severe symptoms). Patients with predominating fatigue symptoms were more likely to have more adaptive personality structure than other patients. Patients with predominating migraine and emotional symptoms differentiated on pre-injury characteristics (i.e., higher SES) and symptom severity (i.e., feeling less normal). Lastly, patients with predominating neurocognitive and emotional symptoms differentiated on personality characteristics (i.e., less adaptive personality structure) and symptom severity (i.e., less severe symptom severity). Taken together, these findings suggest that a complex combination of pre-injury, personality and SRC characteristics may contribute to the constellation of persisting symptoms after SRC.

The results of this study may expose several mechanisms that could influence the configuration of persistent symptoms. One of the most striking findings is that both SRC subgroups with predominating migraine symptoms (i.e., *neurocognitive-migraine* subgroup and *migraine-emotional* subgroup) have poorer outcome in terms of symptoms severity. This aligns with previous research that has identified the presence of migraine symptoms to be associated with relatively long-lasting recovery of SRC [30–32]. Our findings further indicate that among patients with predominating migraine symptoms, patients with lower educational level are more likely to also have predominating neurocognitive symptoms, while patients with higher educational level are more likely to also have predominating emotional symptoms. These findings corroborate with earlier research, showing that symptoms of depression and anxiety after mild TBI are more prevalent among highly educated patients. [33]. Our findings may also reflect that patients with lower educational levels have a smaller cognitive reserve. Cognitive reserve theories suggest that patients with higher education may be able to better compensate the influence of brain injury on daily life functioning with their premorbid higher level of functioning and/or deployment of (more effective) compensation strategies. [34, 35] Since educational level is also subject to cultural stigma [36], other cultural factors such as race and ethnicity might also have an impact on symptom perception and reporting [37]. However, we did not assess these cultural factors and therefore cannot investigate their relevance for our results. Lastly, this study indicates that patients with predominating neurocognitive and migraine symptoms were more likely to have sustained more severe SRC (i.e., presence of loss of consciousness). This supports the idea that persisting post-concussive symptoms might also be accounted for by a biological basis related to injury severity [38]. Together, these findings suggest that educational level and the severity of SRC might importantly influence the configuration of persistent SRC symptoms.

The results of the current study also identified two SRC subgroups with predominating neurocognitive symptoms (i.e., the *neurocognitive-migraine* and *neurocognitive-emotional* subgroups). Noteworthy, 81% of SRC patients with dyslexia were part of one of these subgroups with prevailing neurocognitive symptoms. Indeed, explorative analysis confirms that the prevalence of dyslexia is much higher in patients with predominating neurocognitive symptoms than other patients with persisting SRC (OR = 5.32, *p* = 0.012). This finding may reflect the presence of pre-morbid neurocognitive symptoms in patients with dyslexia, or may reflect that these patients have increased vulnerability for prevailing neurocognitive symptoms after SRC. To our knowledge, this is the first study to show a link between pre-existent dyslexia and prevailing neurocognitive symptoms after SRC, a finding that awaits replication in future studies.

Our findings provided evidence for one subgroup of SRC patients with predominating fatigue-related symptoms. Patients with predominating fatigue-related symptoms were found to have a more adaptive personality structure in general, characterized by the abilities to better able tolerate, use and control emotions and impulses, genuinely care about others, and value someone’s identity and work with others. These results suggest that adaptive personality characteristics may be related to the type of persistent SRC symptoms experienced, where fatigue symptoms are more prevailing than neurocognitive, migraine and vestibular-ocular symptoms. This finding adds to the hypothesis that personality characteristics contribute to the presence and absence of specific persistent post-concussion symptoms [39]. Vice versa, this study showed that having more prominent characteristics of personality disorders (i.e., poor ability to set realistic goals and achieve these goals) is associated with predominating neurocognitive and emotional related symptoms after SRC. This finding is partly supported by a study showing that differences in pre-injury coping (known to be strongly related to personality structure) [40] contributes to the prediction of anxiety and depression symptoms at three months following mild TBI [33]. Taken together, these findings suggest that personality characteristics may influence the configuration of persisting SRC symptoms.

The current results have relevance for concussion management, especially when considering targeted treatment of persisting symptoms. Regarding treatment options, the results indicate that specific pre-injury and SRC characteristics might predispose patients to experiencing specific symptoms in the chronic phase following SRC. Clinicians might use such knowledge when designing an individualized treatment approach from the available and emerging treatment options. For example, SRC patients with higher SES might benefit from additional psychoeducation and psychological treatment, as those patients have a higher risk for emotional disruption after SRC. In addition, specific subgroups of patients with persistent SRC might benefit from other existing and emerging treatments for persisting symptoms (e.g., cognitive behavioral therapy, graded exercise and cervical manual therapy) [41–44]. Future studies may provide valuable information on the role of prevailing symptoms in the efficiency of targeted treatment options for patient subgroups. As for prognostic purposes, results indicate that SRC patients with predominating migraine related symptoms have worse outcome than other patients, which warrants careful prospective monitoring and education of such patients in the early phase of recovery.

This study has some weaknesses. As we used prospectively collected data gathered at a single time point, we could not explore the stability of SRC patient subgroups over time. Relative stability of SRC patient subgroups may be considered important when SRC patient subgroups would be used as a starting point for individualized treatments. It remains to be investigated whether the configuration of dominant symptoms should be regarded as a state or trait of SRC patients. Considering the temporal dynamics of SRC symptoms, we consider it plausible that prevailing symptoms may change over time within patients. If so, it could be suggested that targeted treatment approaches should also flexibly adapt to the evolution of symptom configuration within patients. Furthermore, we used a sample that was confined to the adult age range, whereas research indicates that the relation between SRC symptom subtypes and recovery times may vary across ages [7, 45], which may suggest that SRC symptom subtypes in children might be different from adults. Future research should investigate SRC symptomatology in children specifically. In addition, the sample of our patients may have relatively high educational level as compared to the general population, which may decrease the generalizability of our results to other SRC populations. Finally, injury-related information was retrospectively collected and therefore prone to recall bias. Strengths of this study include the availability of a large group of patients with persisting symptoms of SRC and the use of data-driven methods to explore the heterogeneity of persisting SRC symptoms.

To our best knowledge, this study is the first to provide evidence for persisting symptom subtypes within patients with SRC as well as the existence of patient subgroups with a comparable configuration of predominant symptom subtypes. This evidence may contribute to better understand the heterogeneity in SRCs, especially where more long-lasting forms of SRC exist. In addition, the current study suggests that pre-injury, personality and SRC characteristics may contribute to the emergence of SRC subgroups within patients with persisting symptoms, and that SRC subgroups differ in terms of persisting symptom severity. These results support the idea that subgroups of patients with persisting SRC with a comparable pattern of co-occurring symptom subtypes allow targeted prognosis, clinical management and treatment to optimize recovery of persistent SRC. Future studies should investigate the relationship between SRC subgroups and functional outcome, which may contribute to facilitate optimal combinations of interventions and may be useful to develop novel treatment.

## Data Availability

The datasets generated during and/or analyzed during the current study are available from the corresponding author on reasonable request.
